# Supplementary material for: Early attentional processing and cortical remapping strategies of tactile stimuli in adults with an early and late-onset visual impairment: A cross-sectional study
Source: PLoS One. 2024 Jul 9;19(7):e0306478. doi: 10.1371/journal.pone.0306478 (PMC11232978; doi:10.1371/journal.pone.0306478)
Supplement: Attachment — Submitted filename: Response to reviewers.docx [file pone.0306478.s002.docx]

**RESPONSE TO REVIEWERS**

**Ref.: Ms. No. PONE-D-23-21188**

**Early attentional processing and cortical remapping strategies of tactile stimuli in adults with an early and late-onset visual impairment: a cross-sectional study**

**Plos One**

We thank the editor and the reviewers (1 and 2) for their critical review and valuable comments. We have taken into account all of their recommendations and suggestions. Itemized responses are listed below. All the modifications have been clearly highlighted using the "Track Changes" throughout the manuscript to make its revision easier.

**Academic editor:**

*1. Please ensure that your manuscript meets PLOS ONE's style requirements, including those for file naming.*

We have carefully reviewed the style requirements.

*2. Did you know that depositing data in a repository is associated with up to a 25% citation advantage (https://doi.org/10.1371/journal.pone.0230416)? If you’ve not already done so, consider depositing your raw data in a repository to ensure your work is read, appreciated and cited by the largest possible audience.*

We have deposited data in a repository (<https://doi.org/10.7910/DVN/K7IGAB>).

*3. In your Data Availability statement, you have not specified where the minimal data set underlying the results described in your manuscript can be found. PLOS defines a study's minimal data set as the underlying data used to reach the conclusions drawn in the manuscript and any additional data required to replicate the reported study findings in their entirety.*

We have deposited data in a repository. Relevant URL (<https://doi.org/10.7910/DVN/K7IGAB>) has been included in our revised cover letter.

*4. Please amend either the title on the online submission form (via Edit Submission) or the title in the manuscript so that they are identical.*

This issue has been fixed.

*5. We note that Figure 1 in your submission contain copyrighted images. All PLOS content is published under the Creative Commons Attribution License (CC BY 4.0), which means that the manuscript, images, and Supporting Information files will be freely available online, and any third party is permitted to access, download, copy, distribute, and use these materials in any way, even commercially, with proper attribution.*

Thank you. Figure 1 was created specifically for this study by a collaborator who is listed in the Acknowledgments section. We confirm that this image is not copyrighted by the collaborator or anyone else. You can contact him: Ramon Ahulló. E-mail: ramon.ahullo@uv.es

**Reviewers' comments:**

**Reviewer 1:**

*1. My biggest concern regards the absence or insufficient reporting of source activity statistical analysis. Specifically, the authors report that they used SPM to perform the analysis, but from my understanding the analysis was performed within each group via a one sample test. This approach cannot reveal group differences, though.* *If the authors want to ground an argument in different loci of activation, they should perform a between group comparison with 3 groups, via an F test if they have two-directional hypotheses or a T test if they have one-directional hypotheses. That would actually show if the different groups utilized significantly different cortical patterns of activation to process the stimuli. The regions involved should be discussed and interpreted in such an analysis. In relation to the above, my suggestion would actually be to model the responses to the deviant stimuli as well and include them in the analysis via a 2x3 mixed model design with between subjects factor group and within subjects factor condition. There are several reasons why such an analytical approach would better answer the crucial research question set by the authors:*

*1.1. The early attentional effects on P100 are better evidenced when comparing conditions, not when the P100 is compared against zero, as the inter-individual differences in the amplitude of the ERP (affected by variables such as the scalp thickness etc.) do not allow such an approach to show valid estimation of attentional effects modulation.*

Answer: We appreciate the reviewer's comment and agree with the reviewer's suggestion. In this sense, the differences between the three groups in the increased activity of brain areas were analyzed. We believe that the results are now consistent with the aims of the study. The differences between groups were analyzed using Hotelling’s T2 because, as stated in the literature, the analysis of electroencephalogram signals challenges traditional statistics in several ways (Carbonell et al., 2004; Trujillo-Barreto et al., 2004); thus, Hotelling’s T2 has been proposed for electrophysiological (EEG) neuroimaging analyses (Carbonell et al., 2004) and has previously been used to analyze differences between groups (Requena & López, 2014; Li & Yang, 2022). In addition, some authors have pointed out that Hotelling’s T2 test has a significantly lower type 1 error rate compared to the ANOVA F-test (Lopez, 2019).

Therefore, thanks to the reviewer's comments, some information has been added to the Results and Statistical Analysis sections.

Please, see lines 234-241: The statistically significant sources within each group and between groups were determined using the SPM8 software (The MathWorks Inc., Natick, MA, USA). Thus, an analysis of independent Hotelling’s T2 test against zero was first developed within each group to see the areas of greatest group activity [39,43]. Then, the differences between groups were analyzed to determine the areas that differed the most during the passive tactile recognition task, which computation was based on a voxel by voxel independent Hotelling’s T2 test.

Please, see lines 316-321: At the peak amplitude of P100 during the tactile horizontal line recognition task, the EB group exhibited a higher statistically significant activity compared to the CG in right superior parietal zones. On the other hand, the LB group showed higher statistically significant activation in left orbitofrontal areas compared to the CG. Besides, EB subjects demonstrated statistically significant maximal activation in the left orbitofrontal regions when compared to LB individuals (Figure 4 and Table 3).

*References:*

Carbonell, F., Galán, L., Valdés, P., Worsley, K., Biscay, R. J., Díaz-Comas, L., Bobes, M. A., & Parra, M. (2004). Random field-union intersection tests for EEG/MEG imaging. NeuroImage, 22(1), 268–276. https://doi.org/10.1016/j.neuroimage.2004.01.020

Li X, Yang L, Yan X. An exploratory study of drivers' EEG response during emergent collision avoidance. J Safety Res. 2022 Sep;82:241-250. doi: 10.1016/j.jsr.2022.05.015.

Lopez, Jay, "Effect of Heterogeneity of Variance on the performance of ANOVA F-test and its Alternatives: Simulation Based Study" (2019). University Honors Theses. Paper 788. https://doi.org/10.15760/honors.806

Requena C, López V. Measurable benefits on brain activity from the practice of educational leisure. Front Aging Neurosci. 2014 Mar 11;6:40. doi: 10.3389/fnagi.2014.00040. PMID: 24653699; PMCID: PMC3949114

Taylor J. G. (2007). On the neurodynamics of the creation of consciousness. Cognitive neurodynamics, 1(2), 97–118. https://doi.org/10.1007/s11571-006-9011-8

Trujillo-Barreto, N. J., Aubert-Vázquez, E., & Valdés-Sosa, P. A. (2004). Bayesian model averaging in EEG/MEG imaging. NeuroImage, 21(4), 1300–1319. https://doi.org/10.1016/j.neuroimage.2003.11.008

*1.2. I understand that the authors would like to avoid the deviant condition due to motor artifacts, but if they focus on P100 and not later components, not even preparatory action planning processes would be visible within this time-range (the typical time range is 80-120ms, participants cannot be that fast in evaluating the stimulus and already start preparing for action). Even later components up until 250 ms would be safely analyzed in that way. Overall a significant interaction of condition x group in such an analysis would constitute a valid index of differences in cortical responses related with cross-modal plasticity and early attentional effects.*

Answer: We appreciate the reviewer for his/her comment. In this regard, we agree with the reviewer’s perspective that if we had aimed to assess more complex cognitive processes such as working-memory, an analysis of late components (e.g., those starting at 250 milliseconds) would have been more suitable. However, we intended to observe whether there were changes in early attentional levels, for which the P100 component is an optimal waveform. In fact, as mentioned in the manuscript (lines 346-350), the P100 is considered to provide evidence of the first stages of neuronal processing in the neocortical circuit and it allows the passage of information from lower cortical stages to higher order stages. Therefore, it does not require “preparatory” or voluntary attention from the individual. (Taylor, 2007). Furthermore, P100 is a positive waveform peaking between 80-120 ms (Wynn et al., 2008).

Therefore, thanks to the reviewer's comments, some information explaining P100 has also been added. Please, see lines 114-128: Somatosensory event-related potentials (ERPs) can reveal valuable data about processing spatial tactile information in the cortex [23]. After a stimulus is presented, the electroencephalogram typically exhibits a pattern of oscillations, including the P100 waveform, nestled amidst a series of peaks and troughs. The emergence of P100 activity is believed to occur in primary sensorial cortical areas, offering insight into the initial phases of the neuronal processing within the neocortical circuit [24–27]. There is growing consensus concerning the contribution of P100 waveform in the context of global cognitive processing, notably in situations involving attention. In paradigms in which attention must be directed to a stimulus in space, the ERP P100 has been observed to be modulated by early attentional activity [24]. Spatial attention modulates neural activity as early as 100 ms after stimulus presentation [28,29]. Top-down modulation of cortical activity during the early phases of perceptual processing influences subsequent working-memory management [30]. Thus, neuroplastic changes in VI and early attentional processing can be explored using tools such as ERPs along with quantitative electroencephalography [17].

*References:*

Taylor J. G. (2007). On the neurodynamics of the creation of consciousness. Cognitive neurodynamics, 1(2), 97–118. https://doi.org/10.1007/s11571-006-9011-8

Wynn, J. K., Lee, J., Horan, W. P., & Green, M. F. (2008). Using event related potentials to explore stages of facial affect recognition deficits in schizophrenia. Schizophrenia bulletin, 34(4), 679–687. <https://doi.org/10.1093/schbul/sbn047>

*2. Report of ERP and statistical analysis is not complete. The authors report that the latency is not significantly different amongst groups and provide the corresponding statistical results. Nonetheless, they report that “these waves were similar for the cerebral responses in the three groups”, and similarity is not evaluated somehow, nor the very important measure of ERPs’ amplitude. If a statistically significant effect exists in the amplitude of the P100, it may ground the argumentation provided in the discussion. If no statistically significant effect exist, then basically the authors have an interesting outcome which if not caused by other confounding variables would call for a discussion of why cross-modal plasticity was not present in the study.*

Answer: We would like to thank the reviewer for his/her comment. In this regard, we would like to point out that amplitude is a parameter that reflects the voltage changes per second in an EEG and it is usually measured at each electrode (Bradley & Keil, 2012). Nonetheless, the methodology of our study (lines 220-223) collects the average of the microvolts in all of the electrodes during a time window of 40 ms (-20 ms before and +20 ms after) in order to generate a three-dimensional image that provides us with information about the highest brain activity in each of the brain regions (see Source localization reconstruction section). Thus, Figure 2 and Table 3 show the statistically significant differences in the brain activity in each of the groups. The methodology used in the present study is based on the research objective of observing differences in brain activity in the different groups.

References:

Bradley, M., & Keil, A. (2012). Event-Related Potentials (ERPs). In Encyclopedia of Human Behavior. , 79-85. https://doi.org/10.1016/B978-0-12-375000-6.00154-3.

*3. The main argumentation presented in the discussion expands on possible cross-modal processing differences between the groups. Nonetheless, such differences were not reported statistically in the paper. Hence, the authors would either perform other (and probably more appropriate) analyses to show the effect, or actually discuss more an interpretation of why differences in haptic processing were not observed amongst the groups.*

**Answer:** We thank the reviewer for his/her comment. In this regard, we would like to clarify that we are not evaluating cross-modal plasticity, but rather we are assessing which regions showed maximal activity in early blind, late blind and sighted controls when passively stimulated (not haptic). The fact that we are mentioning cross-modal plasticity is because this is a type of neuroplasticity that arises in people with visual impairment, so that when they are tactilely stimulated, they show increased activity in the occipital region (Lazzouni & Leporé, 2014; Ortiz Alonso et al., 2015). Since the early visually impaired group was the one that showed greater activity in the occipital region, we think that this could be the result of cross-modal plasticity processes. However, reading the wording of the manuscript, we understand that what is written is confusing. Apologies for the confusing wording.

Thanks to the reviewer's comment, some sentences have been modified:

Please, see lines 23-25: Neuroplastic changes appear in people with visual impairment (VI) and they show greater tactile abilities. Improvements in performance could be associated with the development of enhanced early attentional processes based on neuroplasticity.

Please, see lines 96-98: Therefore, this region is a clear example of multimodal spatial information processing [2]. These findings reinforce the hypothesis of substitutive neuroplasticity in people with VI [13].

Please, see lines 106-107: It is suggested that neuroplasticity could be behind the superior tactile skills found in people with VI.

Please, see lines 132-134: We also set out to analyze the maximum brain activity in each group and to compare the brain activity in subjects with early and late-onset VI and a group of sighted individuals.

Please, see lines 414-422: On the other hand, the higher statistically significant activation found in the occipital areas of EB participants in our study, absent in the LB group, suggests that this findings could be due to cross-modal plasticity and its variations, depending on the age of onset blindness. Cross-modal plasticity mainly consists of the brain’s capability to perceive and process stimuli from a sensorial modality different from the original input [13,68,69]. It is an adaptive form of neuroplasticity that appears when there is restriction or loss of a sensorial channel [69]. This has been well-documented in people with VI [12,13,69].

Please, see lines 423-434: It is well-documented in the scientific literature that the occipital cortex remains activated even after blindness acquisition [17,34,70]. Therefore, the activation of occipital structures in EB subjects in our research may be because they mainly utilize the forenamed regions to manage non-visual information. In fact, some studies have claimed that in early-onset visual deprivation, the specialization of hetero-modal areas is preserved, and the ventral and dorsal visual streams are adapted to managing auditory and tactile data [34,71]. Notably, the LOC is a structure that processes non-visual stimuli and, as Voss [71] and Ortiz Alonso [17] stated, it is active in EB individuals, suggesting no need for prior visual experience to recognize tactile objects and shapes in the occipital cortex.

*References:*

Lazzouni, L., & Lepore, F. (2014). Compensatory plasticity: time matters. Frontiers in human neuroscience, 8, 340. https://doi.org/10.3389/fnhum.2014.00340

Ortiz Alonso, T., Santos, J. M., Ortiz Terán, L., Borrego Hernández, M., Poch Broto, J., & de Erausquin, G. A. (2015). Differences in Early Stages of Tactile ERP Temporal Sequence (P100) in Cortical Organization during Passive Tactile Stimulation in Children with Blindness and Controls. PloS one, 10(7), e0124527. https://doi.org/10.1371/journal.pone.0124527

*4. As a minor point, the authors should add a description of P100, how it is modulated by early attentional processes and what are its generators in the introduction so that the reader understands why they decided to use this as an index of the corresponding effects, and whether the interpretation for their results regarding source estimation are reasonably connected to the corresponding literature (the latter should be added in the discussion section).*

**Answer:** We thank the reviewer for his/her valuable comment. Besides, we do agree with his/her opinion. Following his/her comments, some information has been added in the introduction section. We have also added some information throughout the manuscript to explain and discuss the differences between the groups.

Please, see lines 40-47: Compared to the CG, LB individuals showed greater activity in the left orbitofrontal zone, while EB exhibited greater activity in the right superior parietal cortex. The EB had greater activity in the left orbitofrontal region compared to the LB. People with VI may not have faster early attentional processing. EB subjects activate the occipital lobe and right superior parietal cortex during tactile stimulation because of an early lack of visual stimuli and a multimodal information processing. In individuals with LB and EB the orbitofrontal area is activated, suggesting greater emotional processing.

Please, see lines 114-128: Somatosensory event-related potentials (ERPs) can reveal valuable data about processing spatial tactile information in the cortex [23]. After a stimulus is presented, the electroencephalogram typically exhibits a pattern of oscillations, including the P100 waveform, nestled amidst a series of peaks and troughs. The emergence of P100 activity is believed to occur in primary sensorial cortical areas, offering insight into the initial phases of the neuronal processing within the neocortical circuit [24–27]. There is growing consensus concerning the contribution of P100 waveform in the context of global cognitive processing, notably in situations involving attention. In paradigms in which attention must be directed to a stimulus in space, the ERP P100 has been observed to be modulated by early attentional activity [24]. Spatial attention modulates neural activity as early as 100 ms after stimulus presentation [28,29]. Top-down modulation of cortical activity during the early phases of perceptual processing influences subsequent working-memory management [30]. Thus, neuroplastic changes in VI and early attentional processing can be explored using tools such as ERPs along with quantitative electroencephalography [17].

Please, see lines 404-413: A higher statistically significant activity appeared in the anterior cingulate cortex and orbitofrontal areas of LB subjects. The higher activity of the orbitofrontal area was also found in LB individuals when compared to CG. During decision-making, the anterior cingulate cortex plays an essential role in error detection, outcome tracking, and evaluating positive or negative results [63]. Additionally, the anterior cingulate and the orbital cortex are synchronized during an effort-based decision-making task [64]. Burton et al [65] detected activation in areas close to the anterior cingulate cortex in LB people, suggesting the participation of emotional processes during the task. Moreover, the orbitofrontal cortex is involved in reward value and emotional processing. This structure then sends data to the anterior cingulate cortex to learn from the reward or non-reward results [66,67].

Please, see lines 444-451: Finally, our results indicate that, compared to the sighted, the EB group exhibited greater activity in the right superior parietal region. Superior parietal areas are considered as structures that manage a wide range of somatosensory and cognitive processes (e.g., somatosensory integration, mental rotation, motor learning, spatial perception, etc.) [73], and their volume and connectivity are increased in people with EB [74,75]. Our results may be explained because, after early visual loss, the parietal region would consequently acquire the capacity to integrate a greater amount of multimodal information compared to sighted individuals [76]. This augmented capacity could also result from a greater tactile experience [34], as people wit VI typically interact with the external world through their sense of touch [77].

Please, see lines 452-468: It is important to note the greater activity observed in the left orbitofrontal area among participants with EB Compared to LB individuals. The increased activation of this region in the EB group could be linked to the early lack of vision, influencing emotional processes, decision-making, social behavior and cognitive flexibility in a more profound manner. The orbitofrontal cortex plays a crucial role in the ability to adapt flexibly to a dynamically changing environment [78]. In the present study, vertical and horizontal lines where presented in a random sequence, and participants were required to press the button only when the horizontal line appeared, thereby complying with the examiner’s instructions. The maximum activation observed in the orbitofrontal cortex of subjects with EB aligns with the forenamed line detection sequence, as this cortical region is associated with the processing of emotional signals that guide human behavior in tasks where social judgment, reward and punishment are present [66,67]. Additionally, the orbitofrontal cortex is implicated in regulating social behavior, including solving interpersonal problems and fostering social closeness [79]. Vision loss may prompt behavioral adaptations in social interactions (e. g., politeness, humor, avoidance, apologies, etc.) [80], enhanced decision-making [81] and improved detection of ecologically significant emotions [82].

Please, see lines 492-496: On the other hand, LB subjects activated the anterior cingulate cortex and orbitofrontal areas, suggesting emotional processing and a task reward evaluation. The higher activation of the orbitofrontal cortex in LB was also found when compared to sighted individuals.

Please, see lines 498-503: Participants with EB showed maximal activity in the superior parietal region compared to sighted subjects. In addition, when compared to the LB group, individuals with EB exhibited greater activity in the left orbitofrontal region. These findings suggest multimodal information processing, as well as emotional processing associated with social behavior regulation and reward elicitation.

**Reviewer 2:**

*1. Page 5, Methods: (line 109, 114): For early-onset blindness, the authors have considered loss of vision before five years of age. The cutoff age is from 0 to 5 years, with many studies limiting to less than 2 years. What was the rationale?*

**Answer:** We would like to thank the reviewer for his/her comment. Regarding this, the authors would like to clarify that studies whose participants have acquired blindness before the age of 2 years do not justify the rationale for their decision. Instead, our classification is based on what was proposed by other research such as Leporé et al. (2010), Lazzouni and Leporé (2014), which used this classification, and also based on the developmental stages of the V1 anatomical region described by Shiu and Murphy (2018). In the latter article, the authors explain that the most significant development of the V1 region occurs up to 5 years of age. Following the reviewer’s comment, we completed the information in this regard in the manuscript. Please see lines 152-154: in accordance with previous research [33,34]; and based on the developmental stages of the anatomical region of V1 described by Siu and Murphy [35].

*References:*

Lazzouni, L., & Lepore, F. (2014). Compensatory plasticity: time matters. Frontiers in human neuroscience, 8, 340. https://doi.org/10.3389/fnhum.2014.00340

Leporé, N., Voss, P., Lepore, F., Chou, Y. Y., Fortin, M., Gougoux, F., Lee, A. D., Brun, C., Lassonde, M., Madsen, S. K., Toga, A. W., & Thompson, P. M. (2010). Brain structure changes visualized in early- and late-onset blind subjects. NeuroImage, 49(1), 134–140. https://doi.org/10.1016/j.neuroimage.2009.07.048

Siu, C. R., & Murphy, K. M. (2018). The development of human visual cortex and clinical implications. Eye and brain, 10, 25–36. https://doi.org/10.2147/EB.S130893

*2. Page 7, Line 166: Why was the sampling frequency kept at only 1000 Hz?*
**Answer:** We thank the reviewer's comment. Regarding his/her question, we would like to point out that the sampling frequency was determined by what was done in previous studies, such as the study by Ortiz et al. (2011), as well as by the characteristics provided by the EEG device itself. The 1000 Hz provides a waveform construction with more information (1000 samples per second), which is more accurate than if performed, for example, with a sampling frequency of 500 Hz, as performed by Forster et al. (2009).

Thanks to the reviewer's comment, the following information has been added to the manuscript (please, see lines 194-195): Then, as in previous studies [16], the raw signals were digitized using a sampling frequency of 1000 Hz.

*References:*

Forster, B., Sambo, C. F., & Pavone, E. F. (2009). ERP correlates of tactile spatial attention differ under intra- and intermodal conditions. Biological psychology, 82(3), 227–233. <https://doi.org/10.1016/j.biopsycho.2009.08.001>

Ortiz, T., Poch, J., Santos, J. M., Requena, C., Martínez, A. M., Ortiz-Terán, L., Turrero, A., Barcia, J., Nogales, R., Calvo, A., Martínez, J. M., Córdoba, J. L., & Pascual-Leone, A. (2011). Recruitment of occipital cortex during sensory substitution training linked to subjective experience of seeing in people with blindness. PloS one, 6(8), e23264. https://doi.org/10.1371/journal.pone.0023264

*3. Elaborate on the rationale behind epoch between 100ms pre-stimulus to 900ms post.*

**Answer:** We do thank the reviewer for his/her comment. Regarding this, we have rewritten this sentence aiming at clarifying the provided information.

Please, see lines 203-205: A temporal recording window (also known as epochs) lasting 1000 ms was opened, such that the recording started 100 ms before stimulus presentation and continued 900 ms after stimulus presentation.

*4. Page 14, Line 294: “Therefore, these findings suggest the possible existence of a critical period for improving the attentional mechanisms that follow vision loss.” The authors should consider making an informed statement from the literature on this. Since the age of the subjects at the time of study is above 40 years, the inference is incorrect.*

**Answer:** We thank the reviewer for his/her valuable comment. Thanks to this comment, we realized that the two studies using different age cut-offs do not have similar samples or methods to ours, and therefore it is not possible to use them for an effective comparison. We have therefore decided to remove this inference from the manuscript.

*5. Page 16, line 344: Regarding occipital areas, the authors comment “variations occur in cross modal plasticity and depends on the age of onset blindness.”*
*It seems a general statement based on the literature, rather than their conclusion. The authors have not associated the details regarding any association between the two. They may add this information.*

**Answer:** We thank the reviewer’s comment and we fully understand the reviewer. However, we would like to point out that we intended only to provide an explanation as to why activity was found in the occipital cortex in the EB group and to relate it to the numerous studies that found activity in people with EB, not to evaluate cross-modal plasticity. Thanks to their comment, we have tried to remodel some aspects of our wording to make it clearer what we intended to convey and, in addition, we have added a brief explanation as to why we think that maximal activity in the occipital cortex was not found in the LB group.

Please, see lines 414-422: On the other hand, the higher statistically significant activation found in the occipital areas of EB participants in our study, absent in the LB group, suggests that this findings could be due to cross-modal plasticity and its variations, depending on the age of onset blindness. Cross-modal plasticity mainly consists of the brain’s capability to perceive and process stimuli from a sensorial modality different from the original input [13,68,69]. It is an adaptive form of neuroplasticity that appears when there is restriction or loss of a sensorial channel [69]. This has been well-documented in people with VI [12,13,69].

Please, see lines 423-432: It is well-documented in the scientific literature that the occipital cortex remains activated even after blindness acquisition [17,34,70]. Therefore, the activation of occipital structures in EB subjects in our research may be because they mainly utilize the forenamed regions to manage non-visual information. In fact, some studies have claimed that in early-onset visual deprivation, the specialization of hetero-modal areas is preserved, and the ventral and dorsal visual streams are adapted to managing auditory and tactile data [34,71]. Notably, the LOC is a structure that processes non-visual stimuli and, as Voss [71] and Ortiz Alonso [17] stated, it is active in EB individuals, suggesting no need for prior visual experience to recognize tactile objects and shapes in the occipital cortex.

Please, see lines 435-441: In addition, the visual cortex participates in the early recognition of tactile objects (usually between 100-150 ms post-stimulus) [23,72], which is consistent with the present study’s results from the recognition of horizontal lines task. However, a special mention should be made of the fact that no statistical significant higher activity was found in the occipital region in the LB group. According to Lazzouni and Leporé [34], this could be due to the fact that the occipital areas of people with LB remains preserved for visual information management.
