# Supplementary material for: Early attentional processing and cortical remapping strategies of tactile stimuli in adults with an early and late-onset visual impairment: A cross-sectional study
Source: PLoS One. 2024 Jul 9;19(7):e0306478. doi: 10.1371/journal.pone.0306478 (PMC11232978; doi:10.1371/journal.pone.0306478)
Supplement: Attachment — Submitted filename: Response to Reviewers.docx [file pone.0306478.s003.docx]

**ANSWERS TO REVIEWERS**

**Ref.: Ms. No. PONE-D-23-21188**

**Early attentional processing and cortical remapping strategies of tactile stimuli in adults with an early and late-onset visual impairment: a cross-sectional study**

**Plos One**

We thank the editor and the reviewers for their critical review and valuable comments. We have taken into account all of their recommendations and suggestions. Itemized responses are listed below. All the modifications have been clearly highlighted using the "Track Changes" throughout the manuscript to make its revision easier.

Journal Requirements:

Please review your reference list to ensure that it is complete and correct. If you have cited papers that have been retracted, please include the rationale for doing so in the manuscript text, or remove these references and replace them with relevant current references. Any changes to the reference list should be mentioned in the rebuttal letter that accompanies your revised manuscript. If you need to cite a retracted article, indicate the article’s retracted status in the References list and also include a citation and full reference for the retraction notice.

Answer: We have checked the list of references and we have made the following changes:

- The reference number 59 was a commentary on an original article. Consequently, this reference has been changed to cite the original article.
- The reference 68 was an erratum for another article. Consequently, this citation has been withdrawn.

Reviewer #1: I acknowledge the work of the authors in answering my previous concerns, and I find the manuscript to be much improved in the revised version. However, I still have 2 comments:

1. Despite the lower type 1 error rate of the Hotelling’s T2 test, results must still be corrected for multiple comparisons for the interaction effect. Perhaps I missed it, but actually I didn't see any information regarding such a correction for the Hotelling's test in the revised version of the manuscript. Hence, I would like to ask the authors if they performed such a correction or not (if not, it has to be added).

Answer: We would like to thank the reviewer for his/her valuable comment. In response, we would like to clarify that the results were adjusted for multiple comparisons. This information is now included in the manuscript. Our apologies for any confusion this may have caused.

Please, see lines 208-211 (manuscript without track changes): For the between groups comparisons, the statistical analyses were conducted in accordance with the comparisons between the three groups, with the probability of type I error set at 0.017. This was calculated by dividing the established error level (0.05) by the number of comparisons (3).

Please, see line 279 (Table 2): It was considered as statistically significant when p<0.05.

Please, see line 299 (Table 3): It was considered as statistically significant when p<0.017.

2. My second concern regards the fact that the authors did not evaluate the deviant stimuli included in the paradigm. In my opinion since the stimulus was included in the stimulation stream, it had also to be included in the analysis (and it would increase the quality of the research output). I wouldn't like to intervene in the authors' scope, though, so I won't insist on that. Nonetheless, a stronger line of argumentation is needed to justify why it was used in the paradigm, but not in the analysis and interpretation of the data.

Answer: We do thank the reviewer for his/her valuable comment. In this regard, we would like to explain that our objective was to investigate early attentional processing in participants by analysing the P100 wave. This wave is modulated by unconscious and involuntary attention in paradigms where attention must be directed to a particular spatial stimulus (lines 90-92), which justifies the paradigm used in the present study (lines 181-187). In contrast, the analysis of executive responses associated with deviant stimuli is more closely related to cognitive and decision-making processes (Jadhav et al., 2022), which were not the subject of this study. As suggested by the reviewer, this explaining information has been added to the manuscript.

Please, see lines 423-429 (manuscript without track changes): Furthermore, it should be noted that, as previously stated, the P100 wave is modulated by unconscious and involuntary attention in paradigms where the subject must direct attention to a spatial stimulus [24]. However, the analysis of executive responses associated with deviant stimuli is more closely related to cognitive and decision-making processes [82], which were not the subject of this study and therefore this aspect was not examined. Future studies should focus on the analysis of the deviant stimulus to gain insight into the cognitive and decision-making processes of people with VI.

Reference

Jadhav, C., Kamble, P., Mundewadi, S., Jaiswal, N., Mali, S., Ranga, S., Suvvari, T. K., & Rukadikar, A. (2022). Clinical applications of EEG as an excellent tool for event related potentials in psychiatric and neurotic disorders. International journal of physiology, pathophysiology and pharmacology, 14(2), 73–83.

Reviewer #2:

The manuscript has considerably improved, incorporating the suggestion from all the reviewers. Though I still consider the explanation of the role of occipital areas as a little over-interpreted, based on the quality of the manuscript, I consider it as acceptable.

Answer: We would like to thank the reviewer for his/her valuable comment. We will take his/her comment into consideration for future manuscripts and thank he/she for considering our manuscript as acceptable for publication.
